# The relationship of milk expression pattern and lactation outcomes after very premature birth: A cohort study

**DOI:** 10.1371/journal.pone.0307522

**Published:** 2024-07-29

**Authors:** Ilana Levene, Mary Fewtrell, Maria A Quigley, Frances O’Brien

**Affiliations:** 1 Nuffield Department of Public Health, National Perinatal Epidemiology Unit, University of Oxford, Oxford, United Kingdom; 2 Institute of Child Health, University College London, London, United Kingdom; 3 Newborn Care, John Radcliffe Hospital, Oxford, United Kingdom; Sapienza University of Rome: Universita degli Studi di Roma La Sapienza, ITALY

## Abstract

**Introduction:**

Mothers of very premature infants often have difficulties expressing breastmilk, which can cause distress and potential negative impact on infant health. Clinical recommendations on breastmilk expression are extrapolated from term infants’ breastfeeding patterns. This study’s objective was to analyse the association of expressing pattern with lactation outcomes after very premature birth.

**Methods:**

132 participants were recruited after birth between 23+0 and 31+6 weeks’ gestation. Participants recorded the milk expressed in several 24-hour periods in the three weeks after birth.

**Results:**

Expressing frequency was positively associated with 24-hour milk yield, with an adjusted 30.5g increase per expressing session on day four (95% CI 15.7 to 45.3) and 94.4g on day 21 (95% CI 62.7 to 126.2). Expressing ≥8 times per day was associated with higher adjusted milk yield than expressing <6 times (on day four, 146.8g, 95% CI 47.4 to 246.1), but not in comparison to expressing 6–7 times (on day four, 82.1g, 95% CI –25.9 to 190.1). Participants with six months or more prior breastmilk feeding experience had a higher adjusted milk yield than others (on day four, 204.3g, 95% CI 125.2 to 283.3). Night-time (2300–0700 hours) expressing sessions were not associated with increased milk yield after adjustment for time since the prior session. On average, participants who had a longest gap between expressions of less than six hours achieved the UK target of 750g breastmilk, whereas those with a longer gap did not.

**Conclusion:**

Expressing frequency was an important determinant of milk yield. Clinical recommendations to express ≥8 times per day were supported but for some, 6–7 times was sufficient. This was particularly likely for those with six months or more of prior breastmilk feeding experience. A need to express during the night-time hours appeared to be related to minimising the gap between expressions rather than an inherent value of night-time expression.

## Introduction

Premature infants (born at less than 37 weeks’ postmenstrual age; PMA) are at higher risk of partial breastfeeding and premature cessation of breastmilk feeding than term infants [1–3]. This is despite the fact that very premature infants (those born at less than 32 weeks’ PMA) gain particular benefit from maternal breastmilk due to reduction in the serious conditions of necrotising enterocolitis [4] and retinopathy of prematurity [5], and improvement in long term neurodevelopmental outcomes [6,7]. Improving lactation support and increasing exclusive breastmilk for premature infants are research priorities in the area of premature birth in the UK [8] and internationally [9].

World Health Organisation guidance for mothers whose infants are not able to directly breastfeed are to express (extract milk from the breasts mechanically) at least 7 times in 24 hours including at least once at night [10,11]. In the UK this recommendation (from the UNICEF UK Baby Friendly Initiative) is to express 8 to 10 times in 24 hours, with a gap of no longer than six hours at night [12]. This demanding schedule competes with other priorities, including caring for the infant/s, other children and self-care [13]. Qualitative literature emphasises the burden, pressure and mental health impact of frequent expressing [14–17] and this burden can lead to cessation [18,19].

Although the correlation of expressing frequency and milk quantity is well established [20], these precise recommendations are extrapolated from the feeding pattern of term infants [12,21]. There is a single randomised controlled trial of expressing frequency, with a very small sample size [22]. It demonstrated a higher expressed milk yield for mothers advised to express four or more time a day compared to those advised to express three or fewer times, which has little clinical relevance.

Observational studies predominantly show a correlation of expressing frequency and milk yield but most have not assessed the clinically recommended frequency of at least 7–8 expressions per day. One Australian study with 25 participants [23] challenged the recommendations, finding that although expressing more than 6 times per day was associated with higher yield than expressing 4 times per day or less, there was a similar daily milk yield for participants expressing between five and nine times per day. One other small US study analysed the gaps between expressions, reporting that there was no association of milk yield with the longest daily gap between expressions [24]. Given the recommendation to express in the night, it is also important to differentiate between the influence of a long night-time gap between expressions versus a specific need to express in the night-time hours, which no studies have attempted to do. For example, prolactin concentration is higher at night [25,26], which might give particular benefit to night-time expression.

Understanding how to optimise milk synthesis and removal in pump-dependent mothers of preterm infants has been identified as a critical unresolved question [27]. There is low concordance with expressing frequency recommendations in practice, with most studies reporting a median expressing frequency of six times per day and some as low as three [28–32]. Parents want to know more personalised and quantitative information to balance the benefits of frequent expression for lactation outcomes against the risks to physical and mental health [17,33]. For example, in our preparatory study asking parents what questions they have about milk expression, these included “How often to express is actually okay—10 times a day seemed impossible”, “Do you really have to wake up every 3 hours at night” and “how often to express and what time of day is best?” [33] specific quotations personal communication]

This study therefore uses data from an existing cohort of mothers of very premature infants within a randomised controlled trial [34] to study the association of expressing pattern and lactation outcomes. Specifically, the aims of the study are to examine the relationship of expressing frequency, gaps between expressions, circadian pattern and expressing duration with the outcome of contemporaneous expressed milk yield. The results achieve these aims and will add nuance and individualisation to clinical recommendations. Analysis of the relationship between time to first expression and lactation outcomes from this cohort has been published separately [35].

Of note, the words ‘maternal’ and ‘breastmilk’ are used throughout this article. It is acknowledged and respected that some people giving birth may not identify as female, nor choose to use the term breastmilk.

## Methods

### Trial design

This report uses data collected for a randomised controlled trial of a relaxation intervention. A detailed protocol has been published [34]. In brief, 132 people who had given birth to one or two infants between 23+0 and 31+6 weeks’ PMA were recruited in four tertiary and local neonatal units in the United Kingdom. Recruitment took place between 2nd August 2021 and 31st October 2022.

Three of the four neonatal units have UNICEF UK Baby Friendly Initiative level three accreditation, signifying a good level of lactation support. All units provide free hospital grade pumps both at hospital and home and have dedicated infant feeding support staff. Infant feeding support staff had varying qualifications and experience at each site–there was no requirement for specific qualifications such as board certification.

Participants were given a portable scale accurate to 0.1g (Kabalo) and filled in logs each time they expressed milk for 24 hours, on three specific timepoints (day 4, 14 and 21 after birth). The specific gravity of human milk is 1.03 so 1g and 1ml are considered near equivalent [36]. Parents were taught to record the weight of the empty milk container and of the container with milk inside, so that the pure milk weight could be calculated at the analysis stage. Research staff recorded satisfaction with the participants’ ability to use the scale after randomisation and at a day four check-in contact. The participant log template for one site is provided as S1 Fig. Single recording days were selected, over a continuous log, because of parent involvement and recommendations on reducing the burden on participants (described fully elsewhere [33]). Days 4, 14 and 21 specifically were chosen as the recording days because of previous literature examining days 4 and 14 [37,38] and a desire for a later timepoint than day 14.

Other data in the expressing phase of the trial were collected at baseline and day 4, 14 and 21 questionnaires–this included participant-reported time to first expression and participant-reported skin to skin duration. As this was an exploratory analysis of a randomised controlled trial, the number of factors included on questionnaires was limited to the most important potential covariates, to limit participant burden and reduce loss to follow up.

Infant feeding status at 36 weeks’ PMA was assessed by parent text message response and infant medical notes.

The study was approved by the Bloomsbury Research Ethics Committee, London (21/LO/0279) and registered as ISRCTN 16356650. This exploratory analysis was pre-specified. The study was powered in relation to the randomised primary outcome, as described in the published protocol. Applied to the exploratory analysis, the study had 80% power to detect a difference in mean 24-hour milk quantity of 155g between two categories of a binary variable. Power would be higher for continuous variables and lower for variables with more than two categories.

### Outcomes

The outcome variables were 24-hour milk yield and expressing session milk yield. The 24-hour milk yield was defined as the sum of the milk weight expressed at each session in a 24-hour period.

To derive the number of hours from the start of the first logged session of each measurement day to the prior session (where the timing of the previous session was unknown), the final logged session of the same 24-hour period was used as a proxy. This means that an assumption was made that the 24-hour expressing pattern logged on the measurement day was the same as the previous day. For example, if a day 4 log included sessions at 0900, 1200, 1500, 1900 and 0100, the gap to prior session for the 0900 session was assumed to be 8 hours.

### Statistical analysis

Analysis took place on a complete case basis. Analysis of the association between individual-level variables and 24-hour milk yield used linear regression. Analysis of the association between session-level variables and session milk yield used multilevel modelling with individual as a random effect to account for repeated measures. The regression model structures are demonstrated in S2 Fig.

Multivariable adjustment was made for potential sociodemographic and perinatal confounders, and to expressing-related covariates such as time to first expression, the type of expression used and skin to skin contact duration. These potential confounders and important covariates were identified from the literature. Baseline variables were included as candidates for inclusion in multivariable analysis if they showed association with the outcome in univariable analysis with p<0.2 and were retained in the final model if p<0.05 after stepwise removal.

Where multiple timepoints for explanatory variables were available, only measures contemporaneous with the outcome variable were included in statistical models. For example, when the outcome was milk quantity on day four after birth, a variable such as expressing frequency would only be included in the regression model when measured at day four.

Sensitivity analyses were performed to assess potential bias related to outliers and related to the assumptions made in order to assign a gap between expressing sessions to the first logged session of each day (described above). Stata 18.0 was used for analysis.

## Results

### Participant flow

S3 Fig demonstrates the number of participants providing data at each timepoint used in this analysis. At least one 24-hour expressing record was submitted by 108 participants. At the day four assessment, 556 expressing sessions were logged by 103 individuals; at day 14, 577 sessions by 91 individuals and at day 21, 560 sessions by 91 individuals. The majority of missing data occurred at day 14 and 21, for participants who continued in the study but did not provided expressing logs on these days. Researcher observations were that this was predominantly due to participants feeling overwhelmed with caring for their unwell infant and other demands, including expressing milk.

### Participant characteristics

Table 1 shows the participant baseline characteristics. More than half had given birth to extremely premature infants (less than 28 weeks’ PMA) and 70.2% planned to exclusively breastmilk feed (85/121). Overall, 59.5% were of white ethnic background (72/121), 21.4% lived in the least deprived quintile for deprivation (28/131) and 68.3% had at least 19 years of full-time education (82/120).

**Table 1:** Baseline characteristics of the cohort participants.

**Ethnic background, n (%)**	
** White**	72 (60.0)
** Asian or Asian British**	22 (18.3)
** Black, African, Black British or Caribbean**	21 (17.5)
** Mixed or multiple ethnic groups**	3 (2.5)
** Other**	2 (1.7)
** *Prefer not to say/missing***	*12*
**Age (years), mean (SD)**	32.8 (6.3)
**Index of Multiple Deprivation quintile, n (%)**	
** 1 (Least deprived)**	24 (18.3)
** 2**	26 (19.9)
** 3**	22 (16.8)
** 4**	31 (23.7)
** 5 (Most deprived)**	28 (21.4)
** *Missing***	*1*
**Age at leaving full-time education, n (%)**	
** 16 years old or less**	14 (11.7)
** 17 or 18 years old**	24 (20.0)
** 19 years old or more**	82 (68.3)
** *Prefer not to say/missing***	*12*
**Lives with a partner, n (%)**	108 (89.3)
** *Missing***	*11*
**Current smoker, n (%)**	8 (6.6)
** *Missing***	*11*
**Time from birth to first expression of milk (hours), median (IQR)**	6 [2.9 to 12]
** *Missing***	*12*
**Baseline intention for exclusive breastmilk at discharge, n (%)**	85 (70.3)
** *Missing***	*11*
**Mode of birth, n (%)**	
** Vaginal**	57 (43.2)
** Caesarean**	75 (56.8)
**Multiple pregnancy, n (%)**	20 (15.2)
**Primiparous, n (%)**	74 (59.7)
** *Missing***	*8*
**Multipara only, N**	50
** Previous breastmilk feeding experience, n (%)**	
** None**	5 (10.2)
** <6 months**	18 (36.7)
** ≥6 months**	26 (53.1)
** Length of previous breastmilk feeding (weeks), median (IQR)**	38.7 [13.6 to 62.4]
** *Missing***	*1*
**Gestational age at birth (weeks), mean (SD)**	27.8 (2.4)
**Gestational age at birth (weeks); n (%)**	
** 23 to < 26 weeks**	34 (25.8)
** 26 to < 28 weeks**	37 (28.0)
** 28 to < 30 weeks**	29 (22.0)
** 30 to < 32 weeks**	32 (24.2)

### Lactation characteristics

Table 2 shows the expressing pattern characteristics of the cohort for day 4, 14 and 21 at both the 24-hour level and the session level.

**Table 2:** Individual and session-level milk yield and expressing pattern variables on day 4 and 21.

	Day 4			Day 14			Day 21		
	Mean (SD)	Median (IQR)	n	Mean (SD)	Median (IQR)	n	Mean (SD)	Median (IQR)	n
**Individual-level characteristics**									
**Expressed milk yield (grams in 24 hours)**	221.0 (208.3)	154.7 (66.2–295.7)	101	490.6 (345.6)	454.1 (236.7–653)	90	560 (386.4)	490.2 (247.5–830.2)	90
**Expressing frequency (sessions/day)**	4.9 (2.2)	5 (4–7)	103	6.0 (1.9)	6 (5–7)	91	5.7 (2.1)	6 (5–7)	91
**Expressing duration (minutes/day)**	132.4 (84.7)	115 (80–178)	103	161.5 (78.6)	160 (120–204)	91	155.2 (87.7)	150 (110–200)	91
**Longest gap from start of one session to start of prior session (hrs)**	9.9 (5.9)	8 (5.8–11.9)	102	7.4 (4.1)	6.1 (4.7–8.5)	89	7.5 (4.2)	6.5 (5.0–8.5)	87
**Number of direct breastfeeds (per day)**	0.0 (0.2)	0 (0–0)	73	0.1 (0.4)	0 (0–0)	61	0.1 (0.4)	0 (0–0)	51
**Expressing session-level characteristics**									
**Session yield (g)**	44.5 (45.3)	31.3 (15.8–56.2)	501	82.2 (62.3)	71 (36.6–112.7)	537	97.2 (66.4)	86 (47.4–139.4)	519
**Hourly milk yield (g/hr)** *	11.5 (12.4)	8.0 (3.3–15.0)	471	23.1 (18.9)	19.4 (9.8–32.4)	495	27.6 (21.2)	23.6 (13.2–38.5)	479
**Session duration (mins)**	27.6 (13.8)	25 (18.5–30)	556	27.1 (10.9)	26.5 (20–30)	574	27.4 (11.8)	25 (20–30)	560
**Gap from start of session to start of prior session (hrs)**	4.9 (4.1)	3.7 (2.8–5.3)	518	4.1 (2.9)	3.5 (2.8–4.4)	523	4.2 (2.8)	3.4 (2.8–4.6)	501

SD = standard deviation. IQR = interquartile range.

*Milk weight expressed at that session, divided by the number of hours from the start of the session to the start of the prior session.

Few participants achieved UK recommendations on optimal expressing set by UNICEF UK Baby Friendly Initiative (BFI), but achievement of World Health Organisation (WHO) recommendations were more common (Table 3). Fewer than half of participants achieved international recommendations for expressing frequency and longest gap between sessions, on any given day.

**Table 3:** Adherence to international expressing recommendations.

	Timepoint	Achieving the recommendation, n (%)
**UNICEF UK Baby Friendly Initiative recommendations**		
**First expression ≤2 hours from birth**		25/120 (20.8)
**Expressing frequency ≥8 per day**	Day 4	14/103 (13.6)
	Day 14	19/91 (20.9)
	Day 21	15/91 (16.5)
**Longest gap between expressions ≤6 hours**	Day 4	27/102 (26.5)
	Day 14	41/89 (46.1)
	Day 21	36/87 (41.4)
**24-hour milk yield ≥750g**	Day 14	18/90 (20.0)
	Day 21	27/90 (30.0)
**World Health Organisation recommendations**		
**First expression ≤6 hours from birth**		62/120 (51.7)
**Expressing frequency ≥7 per day**	Day 4	29/103 (28.2)
	Day 14	37/91 (40.7)
	Day 21	40/91 (44.0)

At 36 weeks’ PMA, 64.9% (74/114) of participants were giving exclusive breastmilk to their infant/s and 86.0% were giving any breastmilk (98/114). Further description of the cohort’s lactation behaviour is provided in S1 Appendix.

### Relationship of expressing pattern variables with 24-hour milk yield

**Fig 1:**
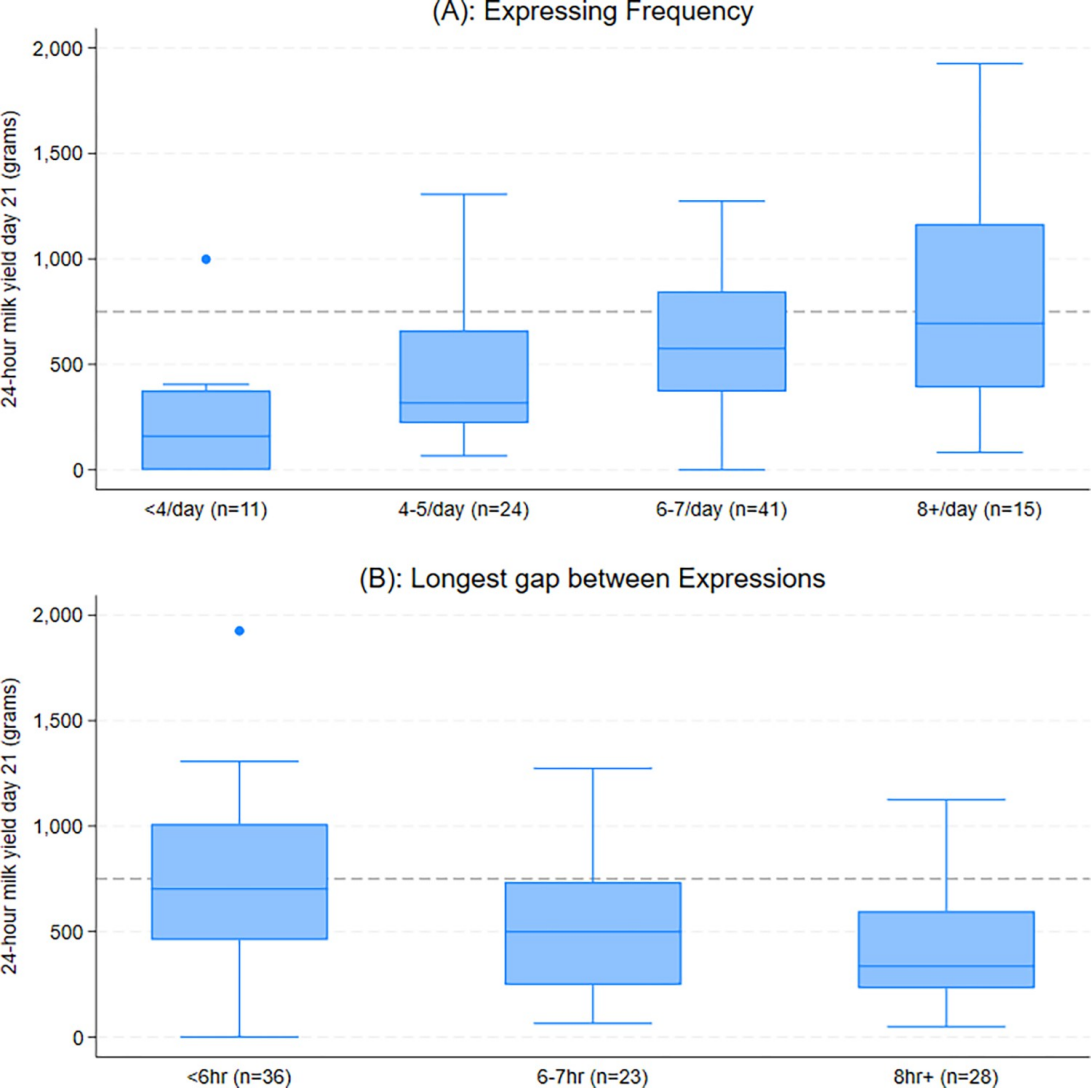
Expressed milk yield on day 21 by categories of expressing frequency (A) and longest gap between expressions (B). 750g is the UNICEF UK Baby Friendly Initiative recommendation for target expressed milk yield by day 10–14 (dashed line). Box plots show median, interquartile range, data range and outliers.

Fig 1 shows the average day 21 milk yield according to expressing frequency and longest gap between expressions, with reference to the UNICEF UK BFI target of 750g. 40% of participants who expressed eight or more times per day achieved this yield target (6/15), in comparison to 37% (15/41) of those expressing 6–7 times, 21% (5/24) of those expressing 4–5 times and 10% (1/10) of those expressing less than four times per day.

In univariable analysis, expressing frequency and longest gap between expressions were each significantly associated with 24-hour milk yield at day four (Table 4). Each increment of expressing frequency was associated with 29.7g higher yield (95% CI 11.8 to 47.7) and each hour of longest gap between expressions was associated with 12.7g lower yield (95% CI -19.5 to -5.9). Expressing frequency accounted for 10% of the variability in 24-hour milk yield, and longest gap for 14% (the R^2^ values). There was no evidence for an association of 24-hour expressing duration with 24-hour yield.

**Table 4:** Crude and adjusted coefficients for the outcome of 24-hour milk yield at day four.

	Unadjusted coefficients (univariable; n = 101 unless marked)		Adjusted coefficients (multivariable; n = 99)*	
	24-hour milk yield in grams (95% CI)	p value	24-hour milk yield in grams (95% CI)	p value
**Expressing pattern variables**				
** Expressing frequency**	**29.7 (11.8 to 47.7)**	**<0.001**	**30.5 (15.7 to 45.3)**	**<0.001**
** Longest gap (per hour)**	**-12.7 (-19.5 to -5.9)**	**<0.001**	-	
** Expressing duration (per hour)**	23.8 (-5.3 to 52.9)	0.11	-	
**Expressing-related potential confounders**				
** First expression ≤6 hours from birth†**	**88.3 (7.1 to 169.4)**	**0.03**	**90.1 (24.9 to 155.3)**	**0.007**
** Electric pump only (compared to manual pump, hand or combination)**	**115.7 (24.0 to 207.4)**	**0.01**	**116.6 (40.9 to 192.3)**	**0.003**
** Simultaneous expression only (compared to single/sequential or combination)**	**107.2 (7.6 to 206.8)**	**0.04**	**112.7 (29.6 to 195.7)**	**0.008**
** Skin to skin contact (per hour)††**	19.2 (-15.6 to 54.1)	0.28	-	
**Baseline potential confounders**				
** Prior breastfeeding ≥6mths (compared to <6mths or primiparous)** ^**¥**^	**202.5 (108.8 to 296.2)**	**<0.001**	**204.3 (125.2 to 283.3)**	**<0.001**
** Caesarean birth**	-44.0 (-127.1 to 39.2)	0.30	-	
** Birth gestation (per week)**	-5.7 (-22.9 to 11.6)	0.52	-	
** Multiple birth**	71.8 (-40.5 to 184.1)	0.21	-	
** Maternal age (per 10yr)**	-26.1 (-97.2 to 45.0)	0.47	-	
** Left full time education ≥18 years†**	28.5 (-63.6 to 120.6)	0.54	-	
** Intention to exclusively breastmilk feed¥**	33.0 (-57.5 to 123.6)	0.47	-	

*all variables in this column were included in the multivariable model. *†n = 99 in univariable regression*. *††n = 89 in univariable regression*. ^*¥*^*n = 100 in univariable regression*.

Because there was a high correlation between expressing frequency and longest gap between expressions, only one of these two variables was used in multivariable analysis (expressing frequency). Expressing frequency was chosen because the longest gap between sessions requires a higher level of derivation from the expressing log than expressing frequency, as described in the methods. The association of expressing frequency with 24-hour milk yield on day four persisted after adjustment for confounders. There was minimal change between the crude and adjusted coefficients for any of the variables included (Table 4). The multivariable model accounted for 43% of the variability in 24-hour milk yield (R^2^).

The same analysis was conducted for milk yield at day 14 and 21 (S1 and S2 Tables). Expressing frequency and longest gap between expressions were also associated with milk yield in unadjusted analysis at day 14 and 21. In addition, expressing duration was associated with milk yield at day 21, but this did not remain significantly associated after adjustment for expressing frequency.

As at day 4, there was minimal change in the coefficient for expressing frequency at day 14 after multivariable adjustment (unadjusted 50.3g, 95% CI 14.4 to 86.2 and adjusted 50.8g, 95% CI 17.8 to 83.9). The coefficient for expressing frequency at day 21 increased after adjustment for prior breastmilk feeding experience, from 85.7g (95% CI 51.2 to 120.3) to 94.4g (95% CI 62.7 to 126.2).

In univariable analysis, expressing frequency accounted for 8% of the variability in 24-hour milk yield at day 14 and 22% of the variability in 24-hour milk yield at day 21. The multivariable models explained 39% and 35% respectively (R^2^).

### Assessing clinical recommendations on expressing pattern

Table 5 shows clinical recommendations for expressing frequency and longest gap between expressions at day 4 and 21. On each day, adjustment was for the variables reported in the multivariable models shown in Table 4, S1 and S2 Tables.

**Table 5:** Adjusted association of expressing pattern in categories related to clinical recommendations, with yield.

	Day 4			Day 14			Day 21		
	24-hour milk yield in grams (95% CI)	p value	n	24-hour milk yield in grams (95% CI)	p value	n	24-hour milk yield in grams (95% CI)	p value	n
**Expressing frequency**									
***Expressing ≥8/day***	*[baseline]*		14	*[baseline]*		19	*[baseline]*		15
***Expressing 6-7/day***	-82.1 (-190.1 to 25.9)	0.14	30	-28.5 (-186.1 to 129.1)	0.72	43	-163.8 (-369.6 to 42.0)	0.12	41
***Expressing <6/day***	**-146.8 (-246.1 to -47.4)**	**0.004**	59	-150.2 (-327.7 to 27.2)	0.10	29	**-402.1 (-615.4 to -188.8)**	**<0.001**	35
**Longest gap between expressions**									
***Longest gap <6 hours***	*[baseline]*		27	*[baseline]*		41	*[baseline]*		36
***Longest gap 6–7 hours***	-41.1 (-137.5 to 55.3)	0.40	23	-100.5 (-254.6 to 53.5)	0.20	20	**-221.0 (-396.8 to -45.2)**	**0.01**	23
***Longest gap ≥8 hours***	**-114.1 (-195.0 to -33.3)**	**0.006**	52	**-152.7 (-292.7 to -12.6)**	**0.03**	28	**-294.6 (-462.3 to -126.9)**	**0.001**	28

Expressing eight or more times per day was associated with significantly higher yield than expressing less than six times per day, but not in comparison to expressing 6–7 times per day, at day 4 and 21. At day 21, a longest gap of less than six hours was associated with higher 24-hour yield than both a longest gap of 6–7 hours and a longest gap of eight hours or more. In contrast, at day four and 14, a longest gap of less than six hours was associated with higher 24-hour yield than a longest gap of eight hours of more, but not the intermediate category of 6–7 hours.

**Fig 2:**
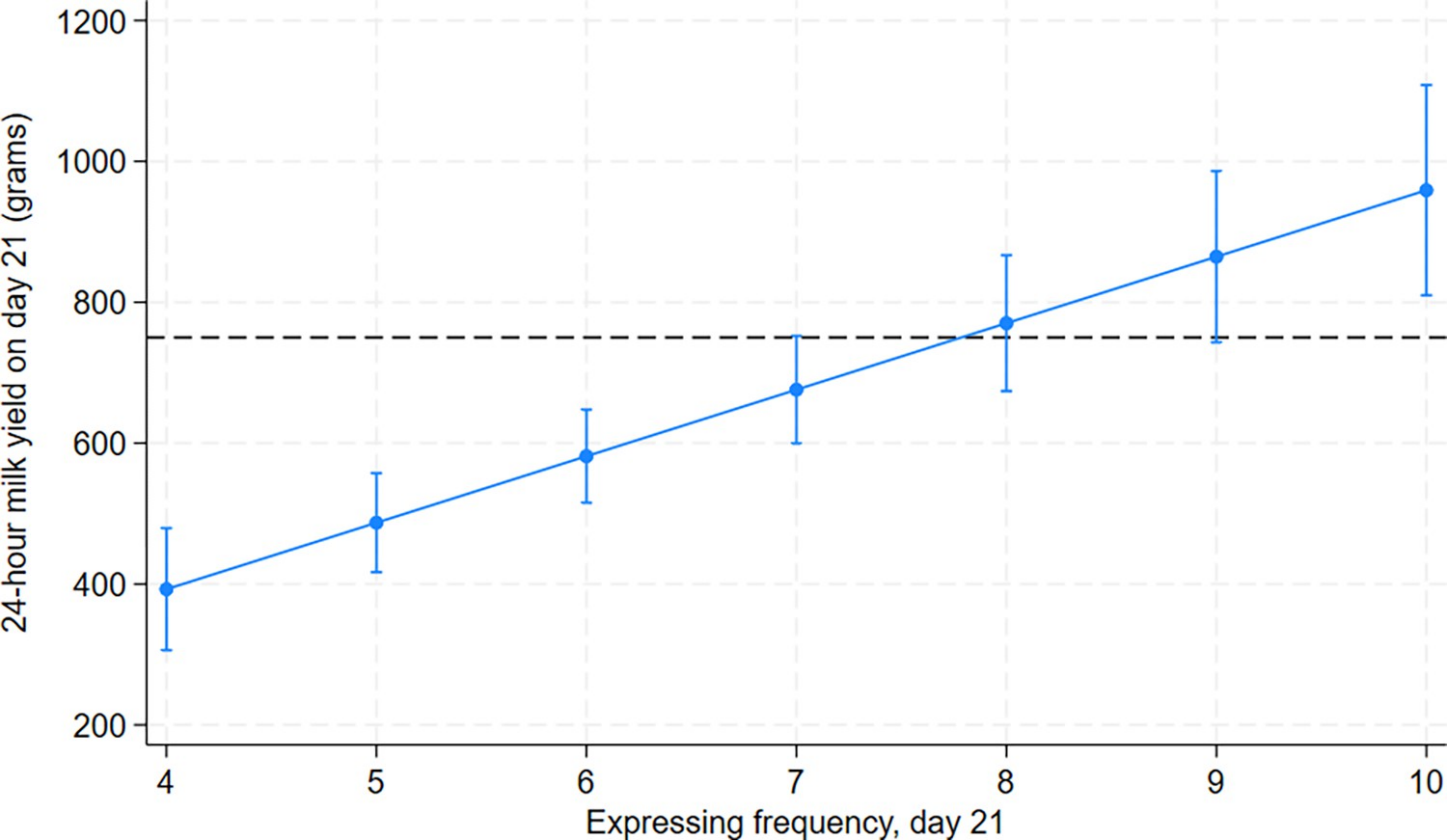
Adjusted 24-hour milk yield by expressing frequency at day 21. Adjusted effect estimate and confidence intervals. 750g is the UNICEF UK Baby Friendly Initiative clinical recommendation for target expressed milk yield by day 10–14 (dashed line).

Clinical recommendations can also be assessed by using the multivariable statistical model to predict adjusted 24-hour milk yield across a range of expressing frequencies. This is shown for day 21 in comparison to the UNICEF UK BFI 24-hour yield target of 750g (Fig 2). The model is adjusted for prior breastfeeding experience. Expressing eight times per day achieved an adjusted 24-hour yield at the target yield, but there is considerable overlap in confidence intervals in the range of 7–9 times per day.

Given the very large effect estimate for the non-modifiable factor of previous breastfeeding experience on milk yield in all the multivariable models presented above, the association of 24-hour yield and expressing pattern is presented according to prior breastfeeding experience in S4 Fig. People with more than six months of prior breastfeeding experience achieved target yield with a much lower expressing frequency than others (on average, at five expressions per day).

### Relationship of session-level expressing pattern variables with session milk yield

The number of hours since the prior session was significantly positively associated with session milk yield at day 14 and 21. Expressing session duration was positively associated with milk yield on all timepoints. After adjustment for the number of hours since prior session, timing of 2300 to 0700 hours (night-time period) was not associated with higher session milk yield at any timepoint (Table 6).

**Table 6:** Crude and adjusted coefficients for the outcome of session milk yield at day 4 and 21.

	Day 4			Day 14			Day 21		
	Unadjusted† coefficient (95% CI)	Adjusted* coefficient (95% CI)	p value	Unadjusted^¥^ coefficient (95% CI)	Adjusted* coefficient (95% CI)	p value	Unadjusted†† coefficient (95% CI)	Adjusted* coefficient (95% CI)	p value
**Time since prior session (hours)**	0.6 (-0.2 to 1.3)	0.8 (-0.0 to 1.6)	0.05	**5.4** **(4.2 to 6.7)**	**5.1** **(3.9 to 6.3)**	**<0.001**	**7.3** **(5.8 to 8.7)**	**6.2** **(4.7 to 7.7)**	**<0.001**
**Session duration (per 10 mins)**	**4.9** **(2.5 to 7.4)**	**5.6** **(3.1 to 8.1)**	**<0.001**	**11.4** **(7.1 to 15.8)**	**10.5** **(6.3 to 14.6)**	**<0.001**	**19.1** **(14.3 to 23.9)**	**15.4** **(10.6 to 20.1)**	**<0.001**
**Session timing**									
** *≥0700 to <1500***	2.9 (-2.3 to 8.1)	2.2 (-3.0 to 7.4)	0.41	**6.9** **(0.5 to 13.4)**	**8.8** **(2.5 to 15.0)**	**0.006**	-1.3 (-9.2 to 6.6)	0.4 (-7.1 to 7.9)	0.92
** *≥1500 to <2300***	1.2 (-4.0 to 6.4)	0.1 (-5.1 to 5.3)	0.97	**-7.8** **(-14.3 to -1.2)**	-0.8 (-7.1 to 5.6)	0.81	**-11.3** **(-19.3 to -3.3)**	-2.5 (-10.2 to 5.2)	0.53
** *≥2300 to <0700***	*[baseline]*			*[baseline]*			*[baseline]*		

†n = 471–501 (101 individuals).

*Adjusted for the listed variables and for method of expression, *simultaneous expression*, *time to first expression*, *prior breastmilk feeding experience and participant age; on day 4 n = 469 (100 individuals)*, *on day 14 n = 495 (88 individuals)*, *on day 21 n = 458 (83 individuals)*. ^¥^n = 495–537 (88 individuals). *††n = 479–519 (86 individuals)*.

### Sensitivity analyses

Sensitivity analyses are described in S1 Appendix. There were some changes to the results related to longest gap between expressing sessions when the assumptions described in the methods section to derive this variable were removed.

## Discussion

### Summary of findings

This study strongly supports the importance [23,39–41] of expressing frequency in relation to 24-hour expressed milk yield. For example, at day 21 there is an adjusted increase in 24-hour yield of 94g per expressing session. However, expressing frequency only explained 8–22% of the variability in milk yield on any one timepoint, so women expressing at the same frequency may have very different milk yields.

UK clinical recommendations to express at least eight times per day were supported because this was associated with achievement of recommended milk yield on average. However, there was wide inter-individual variability and few people expressed at this frequency. Expressing eight or more times a day was not associated with significantly higher 24-hour milk yield than expressing 6–7 times per day, which was closer to the median frequency and therefore more achievable for this cohort. However, participants expressing less than six times per day were unlikely to achieve target yields. This would be useful information for parents who feel that expressing eight times per day is unrealistic or unachievable.

We attempted to analyse the association of longest gap between expressions with 24-hour milk yield. A significant negative association was reported, with milk yield reducing by 30g for each hour increase of the longest gap between expressions on day 21. The clinical recommendation of having a gap no longer than six hours was supported. However, these findings reduced in size and significance when removing certain assumptions required to derive the gap to prior session for the first recorded session of each day. Therefore, this finding should be interpreted with some caution.

The session level analysis reported here was able for the first time to separate two factors associated with the clinical recommendation to express in the middle of the night–the circadian timing and the time since the prior expressing session. The analysis does not support an inherent value to the night-time period, once the time to prior session is accounted for. Although parents would have to express in the night period to keep the longest expressing gap under the recommended six hours, this finding supports flexibility between individuals in exactly when to express, which could take into account their sleep pattern. This is important as sleep interruption is cited as a reason to stop expressing [18].

We have demonstrated a novel exploratory finding that participants with longer prior breastmilk feeding experience (in this study defined as more than six months) express on average less frequently to achieve the same yield as those who are primiparous or have shorter duration of prior breastmilk feeding. This could allow clinicians to individualise expressing recommendations. However, this was not a pre-specified hypothesis and is based on 26 participants with more than six months of breastmilk feeding experience. Therefore this should be interpreted cautiously and requires replication.

### Setting the findings in context

This cohort showed similar expressing frequency to many other cohorts in the literature [23,29,30,32,42], although higher frequency than some [31,43]. Expressed milk yield was broadly similar to previous cohorts [39,40,42,43] although notably lower than two small studies that looked in depth at expressing dynamics [23,44]. This may suggest that our study recruited a broader range of participants, not just the most motivated.

The sample is not representative of very premature infants in general in the UK–it was predominantly recruited from Southern English units, which tend to have higher breastmilk prevalence at discharge home [45], and three out of four neonatal units are level three BFI accredited, suggesting a better than average level of lactation support. This likely explains why the prevalence of any breastmilk at 36 weeks’ PMA in this report (86%) was much higher than the national prevalence reported at discharge home in the National Neonatal Audit Programme (60% in 2020) [46]. Although this may reduce generalisability, it is useful for analysis of the relationship of expressing behaviour with breastfeeding outcomes to study a cohort with high intention to breastmilk feed.

The positive association of expressing frequency and milk yield we have found is consistent with most other studies [23,31,32,39,41,47–51]. One previous study showed a smaller adjusted increase in 24-hour yield of 25ml per expressing session (at average day 14) [50]. Only one study is known to have reported on the longest gap between expressions previously, finding no association with milk yield at two or four weeks after birth [24]. Mean longest gap was shorter in that report (6.5 hours) than seen here (7.5 to 10 hours depending on the timepoint).

In the NICU context, two previous studies with mothers of very preterm infants have shown a similar magnitude of increase in milk yield with longer previous breastfeeding experience—higher milk yield by 222ml/day [52] and 22–25% greater milk yield at week one and four [53]. One study with mixed gestation NICU mothers showed a median difference in duration of exclusive breastfeeding of one month for those with any previous breastfeeding experience [54]. In contrast, one study showed no correlation of previous breastfeeding experience with ability to express at least 500g per day by week 5 [37].

It is possible that at least part of this finding is due to confounding, whereby people with longer length of breastmilk feeding are more motivated, have higher self-efficacy [55] and may put in place unmeasured supportive behaviours to achieve higher milk yield. However, there could also be an element of physiological difference in the breast tissue of someone who has experience prolonged previous lactation. In support of this idea, one study [56] has shown that milk production per unit of breast tissue is much higher after six months of lactation (for example 4g/ml at nine months compared to 2g/ml in the first six months).

It is well established in mice that mammary epithelial cells show epigenetic changes after the first pregnancy/lactation cycle that causes earlier activation of milk-related genes for subsequent pregnancy/lactation cycles [57,58]. In multiparous rats, there is increased responsiveness to prolactin and therefore a lower circulating prolactin concentration [59]. A similar phenomenon has been seen in two human studies. One showed lower circulating prolactin in the first four days after birth in multiparous women, but higher infant milk intake [60]. The second showed increased milk yield at day seven after the woman’s second birth in comparison to their first birth, despite shorter total duration of feeding time [61]. In addition, increased length of prior breastfeeding has been linked with greater epithelial area and increased expression of progesterone receptors in clinical breast tissue samples, even a considerable time after cessation of lactation [62]

### Strengths and limitations

The strengths of this study are the broad population of included participants, in terms of sociodemographic variables as well as expressing behaviour. The inclusion of individual and session level variables across time has yielded novel findings and will help clinicians to personalise the advice they offer to families.

The most significant limitation of the study is the sample size, including some loss to follow up in expressing log submission. Because of the increased loss to follow up at day 14 and 21, the findings at day 4 likely have a lower risk of bias than those at day 14 and 21. A further limitation of the study is the reliance on participant-measured data on expressing pattern and yield, which could affect data accuracy; and the restriction of data collection to single days rather than a continuous log. Single day logs meant that some assumptions were required to derive the gap to prior session for the first expressing session of the day, which had a significant impact on the analysis conclusions related to the longest gap between expressions. The characteristics of the first expression of the day were significantly different from others so it is unclear whether the impact on the analysis was because of bias in the derivation assumption or because these expressing sessions have an important and differential influence on the outcomes. In addition, some expressing dynamics may relate better to cumulative expressing behaviours, which may not be adequately represented by single day logs.

Both elements of trial design (participant measurement and single day collection timepoints) were chosen after extensive parent involvement, to reduce interference and burden for participants at a stressful time [33]. This may have resulted in the broader representation of expressing behaviour and lactation outcomes than seen in other detailed studies [23,44] and may be considered a trade-off when studying milk expression at this stressful time.

There were several variables that could not be included in this analysis that are known to be important for expressed milk outcomes; most notably maternal factors such as chorioamnionitis [38,63], assisted reproduction [63,64] and obesity [63,65]. This could lead to residual confounding.

## Conclusions

Overall, the data in this study provides much needed evidence related to current clinical recommendations on expressing patterns after very preterm birth. UK and international recommendations on expressing frequency and longest gap between expressions were supported, but there are significant limitations to average-based recommendations in a population with such wide inter-individual variability.

Families can be counselled that expressing pattern is only one determinant of milk yield, and that those with an otherwise good risk profile for lactation may be able to express at a lower frequency than the general recommendation. A change in emphasis can be suggested related to clinical advice on night-time expression, which appears to be valuable in relation to limiting the gap between expressions rather than because of its specific circadian timing.

More work is required to replicate the exploratory finding of the importance of prior breastfeeding experience and identify further variables that explain the residual variability. This would help families understand their risk factors in relation to milk yield, bringing potential for individualisation of recommendations and also might identify potential further targets for intervention.

## Supporting information

S1 Fig Template for the participant expressing log at one of the research sites. (PDF)

S2 Fig Diagrammatic representation of one level and multilevel modelling used in this analysis for individual level and session level analysis of milk yield. (PNG)

S3 Fig Participant and data flow diagram. (JPG)

S4 Fig (A): Scatter graph of expressing frequency and 24-hour milk yield on day 21 according to breastfeeding experience. (B) Modelled 24-hour milk yield on day 21. Unadjusted linear regression lines are shown in (A). (B) includes a statistical interaction between expressing frequency and breastfeeding experience.
(JPG)

S1 Appendix Supplementary text with associated figures. (DOCX)

S1 Table Crude and adjusted coefficients for the outcome of 24-hour milk yield at day 14. (DOCX)

S2 Table Crude and adjusted coefficients for the outcome of 24-hour milk yield at day 21. (DOCX)
