# Supplementary material for: The relationship of milk expression pattern and lactation outcomes after very premature birth: A cohort study
Source: PLoS One. 2024 Jul 29;19(7):e0307522. doi: 10.1371/journal.pone.0307522 (PMC11285974; doi:10.1371/journal.pone.0307522)
Supplement: S1 Fig — (PDF) [file pone.0307522.s001.pdf]

EXPRESS

EXpressing in PREmaturity - Simple interventionS

Chief Investigator: Ilana Levene

Email: ilana.levene@ndph.ox.ac.uk

National Perinatal Epidemiology Unit, University of Oxford,
Old Road Campus, Oxford. OX3 7LF

Date: D D / M M / Y Y **Study ID:** ☐☐☐☐☐

Day 4 ☐ **Day 14** ☐ **Day21** ☐ **32 weeks' postmenstrual age** ☐

24 hour expressing log

Please log each time you express milk today, starting from when you wake up for the day and continuing for 24 hours after the first time you express (for example, if you first express at 09:30 please log every time you express until 09:29 the following day).

If you are no longer expressing milk, please tick this box ☐

If you plan to continue expressing milk but you have not expressed at all in this 24 hour period, please tick this box ☐

If you are using the standard expressing containers from John Radcliffe Hospital neonatal unit you can use the following weights when the log asks for the weight of the empty container. Large 130ml bottle (with lid) =22g. Medium 100ml bottle (with lid) = 18.5g. Colostrum syringe (with cap) = 4g. If you are using a different container, including if you move to a different hospital with different bottles, then please weigh it when it's empty.

When did you start expressing?		Method			How long did you express for? *(minutes)*	Weight of milk and container - right breast *(with lid on; grams)*	Weight of milk and container - left breast *(with lid on; grams)*	Weight of empty container *(with lid on; grams)*
Date	Time	Hand	Manual pump	Electric pump				
D D / M M / Y Y	h h : m m 24hr	☐	☐	☐	☐☐	☐☐.☐	☐☐.☐	☐☐.☐
D D / M M / Y Y	h h : m m 24hr	☐	☐	☐	☐☐	☐☐.☐	☐☐.☐	☐☐.☐
D D / M M / Y Y	h h : m m 24hr	☐	☐	☐	☐☐	☐☐.☐	☐☐.☐	☐☐.☐
D D / M M / Y Y	h h : m m 24hr	☐	☐	☐	☐☐	☐☐.☐	☐☐.☐	☐☐.☐
D D / M M / Y Y	h h : m m 24hr	☐	☐	☐	☐☐	☐☐.☐	☐☐.☐	☐☐.☐
D D / M M / Y Y	h h : m m 24hr	☐	☐	☐	☐☐	☐☐.☐	☐☐.☐	☐☐.☐
D D / M M / Y Y	h h : m m 24hr	☐	☐	☐	☐☐	☐☐.☐	☐☐.☐	☐☐.☐

When did you start expressing?		Method			How long did you express for? *(minutes)*	Weight of milk and container - right breast *(with lid on; grams)*	Weight of milk and container - left breast *(with lid on; grams)*	Weight of empty container *(with lid on; grams)*
Date	Time	Hand	Manual pump	Electric pump				
DD/MM/YY	hh:mm 24hr					.	.	.
DD/MM/YY	hh:mm 24hr					.	.	.
DD/MM/YY	hh:mm 24hr					.	.	.
DD/MM/YY	hh:mm 24hr					.	.	.
DD/MM/YY	hh:mm 24hr					.	.	.
DD/MM/YY	hh:mm 24hr					.	.	.
DD/MM/YY	hh:mm 24hr					.	.	.
DD/MM/YY	hh:mm 24hr					.	.	.
DD/MM/YY	hh:mm 24hr					.	.	.
DD/MM/YY	hh:mm 24hr					.	.	.
DD/MM/YY	hh:mm 24hr					.	.	.

Thank you for taking the time to fill in this expressing log. If you would like more support with expressing, please talk to your neonatal nurse or the infant feeding team if there is one. There is more useful information here:

https://www.bliss.org.uk/parents/about-your-baby/feeding/expressing

https://www.bestbeginnings.org.uk/small-wonders

If you would like more support with your mental health and how you are feeling please talk to your neonatal nurse or the psychologist if there is one. There is more useful information here:

https://www.bliss.org.uk/parents/support/impact-mental-health-premature-sick-baby/getting-support-mental-health

EXPRESS Trial, NPEU CTU, National Perinatal Epidemiology Unit, Nuffield Department of Population Health, University of Oxford, Old Road Campus, Oxford OX3 7LF

01865 289751 01865 289740 express@npeu.ox.ac.uk www.npeu.ox.ac.uk/express

FUNDED BY NIHR | National Institute for Health Research

EXPRESS is funded by the National Institute for Health Research (NIHR). The views expressed are those of the author(s) and not necessarily those of the NIHR or the Department of Health and Social Care.

NPEU Clinical Trials Unit   UNIVERSITY OF OXFORD
