# Supplementary figures and images for: The relationship of milk expression pattern and lactation outcomes after very premature birth: A cohort study
Source: PLoS One. 2024 Jul 29;19(7):e0307522. doi: 10.1371/journal.pone.0307522 (PMC11285974; doi:10.1371/journal.pone.0307522)

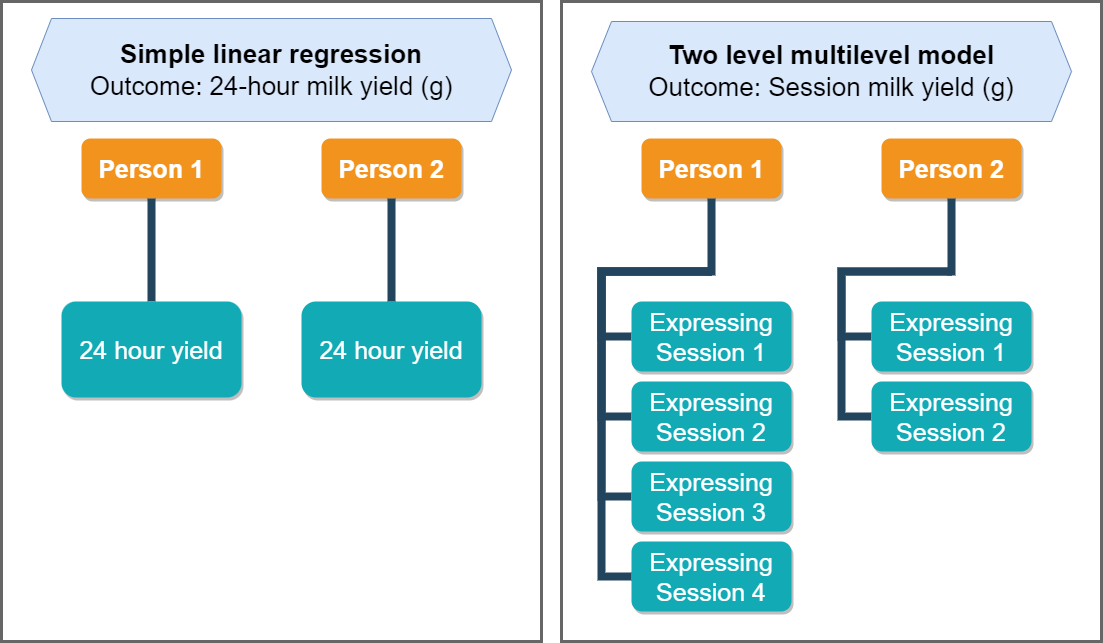

Supplement: S2 Fig — (PNG) [file pone.0307522.s002.png]

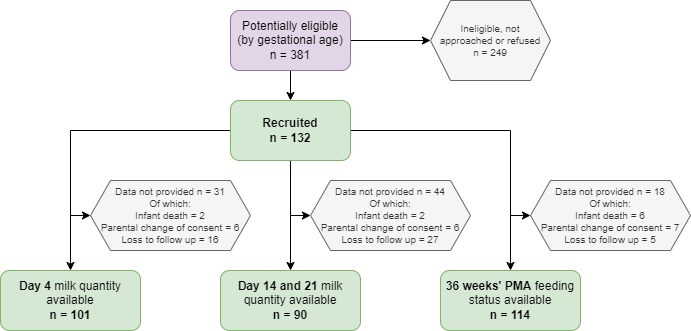

Supplement: S3 Fig — (JPG) [file pone.0307522.s003.jpg]

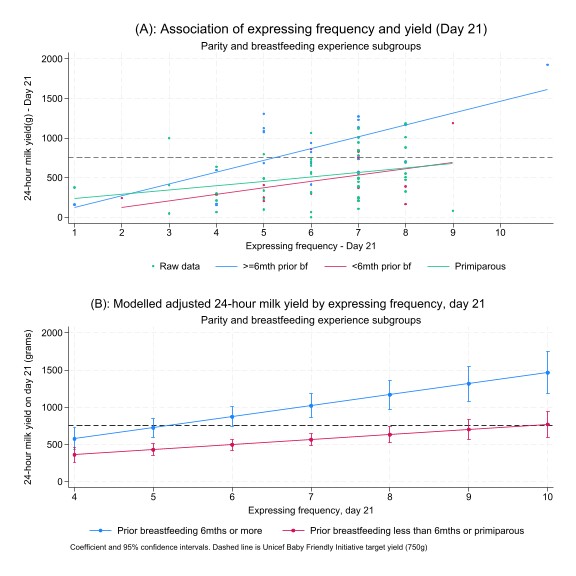

Supplement: S4 Fig — (A): Scatter graph of expressing frequency and 24-hour milk yield on day 21 according to breastfeeding experience. (B) Modelled 24-hour milk yield on day 21. Unadjusted linear regression lines are shown in (A). (B) includes a statistical interaction between expressing frequency and breastfeeding experience. (JPG) [file pone.0307522.s004.jpg]
