# Supplementary material for: The relationship of milk expression pattern and lactation outcomes after very premature birth: A cohort study
Source: PLoS One. 2024 Jul 29;19(7):e0307522. doi: 10.1371/journal.pone.0307522 (PMC11285974; doi:10.1371/journal.pone.0307522)
Supplement: S1 Appendix — (DOCX) [file pone.0307522.s005.docx]

**Further lactation characteristics of the cohort**

Participants were predominantly using an electric pump (75% at day four and 87% at day 21) and expressing both breasts simultaneously (79% on day four and 84% on day 21). The majority of individuals expressed at least once in the night-time period (2300 to 0700); 76% on day four and 84% on day 21.

On day four, 47.9% participants reported any skin-to-skin contact with their infant/s (46/96), which increased to 77.7% by day 21 (66/85). Median duration of skin-to-skin contact was 1 hour on day four and 2 hours on day 21.

24-hour milk yield increased over time from median 154.7g on day four to 490.2g on day 21, and measurements were strongly correlated between days (shown below in panel B). This correlation was strongest between day 14 and 21 (r=0.90, p<0.001).

Expressing frequency was strongly correlated with the longest gap between expressions (day 4 r=-0.87, day 14 r=-0.74 and day 21 r=-0.78, p<0.001). This relationship is shown below in panel C.

1. **Box plot of 24-hour milk yield over time with median values identified**
2. **Scatter plot of 24-hour milk yield on consecutive timepoints, with linear regression line and Pearson correlation coefficient**
3. **Scatter plot of expressing frequency and longest gap between sessions on day 21, with linear regression line**

The trajectories of milk yield were highly variable between individuals (shown below). Although there was a linear increase over time on average, a third of the participants had lower milk yield at day 21 than at day 14 (24/83, 28.9%).

**Individual milk yield trajectories, with mean trajectory displayed in red**

**Sensitivity analyses**

Removing one participant who was a high outlier for both 24-hour milk yield and expressing frequency did not change the analysis conclusions.

Removing the assumptions required to assign a gap to prior session to the first logged session of the measurement period (described in the methods section) reduced the association seen between the longest gap between expressions and 24-hour yield. On day four, the coefficient for longest gap reduced to -11.0g (95% CI -28.1 to 6.2, n = 92) and on day 21, to -15.3g (-52.9 to 22.4, n = 84). On day 21, expressing with a longest gap of less than six hours was no longer significantly associated with higher 24-hour adjusted yield compared to 6-7 hours or more than 7 hours.

The affected first sessions represented the longest gap in the 24-hour period for 47.1% of day four logs (48/102) and 37.9% (33/87) of day 21 logs. Excluding these sessions leads to more participants being classified as having a longest gap between expressing sessions of 6 hours or less. On day four this would rise from 27/102 (26.5%) with the derivation assumption to 50/92 (54.4%) without the assumption. On day 21 it would rise from 37/87 (42.5%) to 58/84 (69.1%). First logged sessions had significantly greater milk yield on day 21 (median difference 34.6g, p=0.001) and significantly greater gap to prior session on both days (on day 21 median difference 3.0 hours, p<0.001), than other sessions.
