# Supplementary material for: The relationship of milk expression pattern and lactation outcomes after very premature birth: A cohort study
Source: PLoS One. 2024 Jul 29;19(7):e0307522. doi: 10.1371/journal.pone.0307522 (PMC11285974; doi:10.1371/journal.pone.0307522)
Supplement: S1 Table — (DOCX) [file pone.0307522.s006.docx]

|  | Unadjusted coefficients  (univariable; n = 90 unless marked) | | Adjusted coefficients  (multivariable; n=89) | |
| --- | --- | --- | --- | --- |
|  | **24-hour milk yield in grams (95% CI)** | **p value** | **24-hour milk yield in grams (95% CI)** | **p value** |
| Key expressing variables |  |  |  |  |
| Expressing frequency | **50.3 (14.4 to 86.2)** | **0.007** | **50.8 (17.8 to 83.9)** | **0.003** |
| Longest gap (per hour)† | **-23.3 (-40.6 to -5.9)** | **0.009** |  |  |
| Expressing duration (per hour) | 47.2 (-7.7 to 102.1) | 0.09 |  |  |
| Expressing-related potential confounders |  |  |  |  |
| First expression ≤6 hours from birth†† | **155.7 (12.2 to 299.3)** | **0.03** |  |  |
| Electric pump only (compared to manual pump, hand or combination)†† | 160.1 (-92.1 to 412.4) | 0.21 |  |  |
| Simultaneous expression only (compared to single/sequential or combination)†† | **318.5 (108.0 to 529.0)** | **0.003** | **206.8 (24.8 to 388.8)** | **0.03** |
| Skin to skin contact (per hour)* | **63.0 (20.0 to 106.1)** | **0.005** |  |  |
| Baseline potential confounders |  | |  |  |
| Prior breastfeeding ≥6mths (compared to <6mths or primiparous) | **360.1 (198.8 to 521.4)** | **<0.001** | **367.9 (219.4 to 516.4)** | **<0.001** |
| Caesarean birth | -90.2 (-239.1 to 58.8) | 0.23 |  |  |
| Birth gestation (per week) | -9.1 (-40.3 to 22.1) | 0.56 |  |  |
| Multiple birth | **276.2 (84.0 to 468.4)** | **0.005** | **224.9 (62.1 to 387.7)** | **0.007** |
| Maternal age (per 10yr) | 30.1 (-126.9 to 187.1) | 0.71 |  |  |
| Left full time education ≥18 years†† | **190.5 (30.2 to 350.8)** | **0.02** |  |  |
| Intention to exclusively breastmilk feed | 59.8 (-100.4 to 219.9) | 0.46 |  |  |

*†n=88 in univariable regression. ††n=89 in univariable regression, *n=82 in univariable regression.*
