# Supplementary material for: The relationship of milk expression pattern and lactation outcomes after very premature birth: A cohort study
Source: PLoS One. 2024 Jul 29;19(7):e0307522. doi: 10.1371/journal.pone.0307522 (PMC11285974; doi:10.1371/journal.pone.0307522)
Supplement: S2 Table — (DOCX) [file pone.0307522.s007.docx]

|  | Unadjusted coefficients  (univariable; n = 90 unless marked) | | Adjusted coefficients  (multivariable; n=90) | |
| --- | --- | --- | --- | --- |
|  | **24-hour milk yield in grams (95% CI)** | **p value** | **24-hour milk yield in grams (95% CI)** | **p value** |
| Key expressing variables |  |  |  |  |
| Expressing frequency | **85.7 (51.2 to 120.3)** | **<0.001** | **94.4 (62.7 to 126.2)** | **<0.001** |
| Longest gap (per hour)† | **-29.7 (-50.4 to -9.1)** | **0.005** | - |  |
| Expressing duration (per hour) | **88.7 (35.4 to 141.9)** | **0.001** | - |  |
| Expressing-related potential confounders |  |  |  |  |
| First expression ≤6 hours from birth†† | 73.6 (-91.4 to 238.7) | 0.38 | **-** |  |
| Electric pump only (compared to manual pump, hand or combination)† | 108.2 (-134.6 to 351.1) | 0.38 | **-** |  |
| Simultaneous expression only (compared to single/sequential or combination) ¥ | 144.8 (-73.8 to 363.4) | 0.19 | - |  |
| Skin to skin contact (per hour)* | 41.4 (-4.5 to 87.4) | 0.08 | - |  |
| Baseline potential confounders |  | |  |  |
| Prior breastfeeding ≥6mths (compared to <6mths or primiparous) | **278.5 (98.5 to 458.4)** | **0.003** | **335.9 (181.8 to 490.1)** | **<0.001** |
| Caesarean birth | 32.4 (-133.6 to 198.4) | 0.70 | - |  |
| Birth gestation (per week) | -7.6 (-42.0 to 26.7) | 0.66 | - |  |
| Multiple birth | 192.5 (-28.3 to 413.4) | 0.09 | - |  |
| Maternal age (per 10yr) | 47.9 (-112.6 to 208.4) | 0.56 | - |  |
| Left full time education ≥18 years†† | **301.5 (132.5 to 470.5)** | **0.001** | - |  |
| Intention to exclusively breastmilk feed | 144.0 (-35.2 to 323.1) | 0.11 | - |  |

*†n=86 in univariable regression. ††n=89 in univariable regression, ¥n = 87 in univariable regression. *n=82 in univariable regression.*
